# Investigation of the polyvinyl alcohol stabilization mechanism and adsorption properties on the surface of ternary mixed nanooxide AST 50 (Al_2_O_3_–SiO_2_–TiO_2_)

**DOI:** 10.1007/s11051-014-2831-2

**Published:** 2015-01-11

**Authors:** Małgorzata Wiśniewska, Iwona Ostolska, Katarzyna Szewczuk-Karpisz, Stanisław Chibowski, Konrad Terpiłowski, Vladimir Moiseevich Gun’ko, Vladimir Iljich Zarko

**Affiliations:** 1 Department of Radiochemistry and Colloids Chemistry, Faculty of Chemistry, Maria Curie-Sklodowska University, M. Curie-Sklodowska Sq. 3, 20-031 Lublin, Poland; 2 Department of Physical Chemistry – Interfacial Phenomena, Faculty of Chemistry, Maria Curie-Sklodowska University, M. Curie-Sklodowska Sq. 3, 20-031 Lublin, Poland; 3 Institute of Surface Chemistry, National Academy of Sciences in Ukraine, 17 General Naumov Street, Kiev, 03164 Ukraine

**Keywords:** Mixed fumed oxides, Alumina–silica–titania, Suspensions stability, Polymer adsorption, Zeta potential, Potentiometric titration, Poly(vinyl alcohol)

## Abstract

A new adsorbent consisting of fumed, mixed alumina, silica, and titania in various proportions (AST 50) was investigated. The studied material was prepared by chemical vapor deposition method. The diameter of AST 50 primary particles was equal to about 51 nm which denotes that it can be classified as a nanomaterial. In the presented paper, the adsorption properties of polyvinyl alcohol on the ternary oxide were investigated. The polymer macromolecules were characterized by two different molecular weights and degree of hydrolysis. The polymer adsorption reaches the maximum at pH 3 and decreases with the solution pH rise. The reduction of the adsorbed PVA macromolecules is related to the electrostatic repulsion forces occurring in the studied system. The AST 50 point of zero charge (pH_pzc_) obtained from the potentiometric titration is equal to 4.7. Due to the nonionic character of the analyzed macromolecular compound, the polymer attendance has an insignificant effect on the AST 50 surface charge density. In the case of the adsorbent particles zeta potential, the obtained dependencies are different in the absence and presence of PVA. The shift of the slipping plane and displacement of the counter-ions from Stern layer by the adsorbed polymer chains have the greatest effect on the *ζ* potential value. The stability measurements indicate that the AST 50 suspensions in the presence of the background electrolyte at pH 3 and 6 are unstable. In turn, in an alkaline medium the mixed oxide suspensions exhibit the highest durability, which is a result of a large number of the negative charges on the AST 50 surface. The addition of PVA 100 significantly improves the suspension stability at pH 3 and 6; at higher pH value, the polymer presence does not influence the system durability. It is related to the steric and electrosteric stabilization of the colloidal particles by the adsorbed polyvinyl alcohol macromolecules.

## Introduction

Polymer adsorption process at the solid–liquid interface has numerous applications in various branches of the industry and human activity (Mauer 2004; Liufu et al. 2005; Farrokhpay 2009; Moody 1992; Amjad 2002; Grządka 2011). Macromolecular compounds have considerable influence on the colloidal system stability. One of the substances most commonly used in the industry is polyvinyl alcohol—PVA. This nonionic synthetical polymer exhibits good solubility in water and excellent biodegradability. These features enable application of PVA as a emulsifier, colloidal particles stabilizer, adhesive, and coating agent in the textile and paper industries. Biocompatibility, biodegradability, and a lack of toxicity favor the broad utilization of PVA in medicine as well as in the cosmetic and pharmaceutic industries (Cai and Gupta 2002; Briscoe et al. 2000).

Technical development and improvement of macromolecular substances adsorption processes necessitate the search for new materials and methods for their synthesis. An outstanding group of adsorbents are mixed oxides (named also nanooxides or ternary oxides), which include compounds of the suitable metals such as silicon, aluminum, and titanium (in various proportions). These materials possess significantly different properties and structure compared to the starting substrates. The investigations including the determination of the ternary oxides suspensions behavior are very important in relation to its unusual properties resulting from the surface structure. In addition, it is possible to obtain the nanooxides of the desired type, amount, and distribution of the surface active groups which provide appropriate binding of simple ions or polymer macromolecules. Thus, these adsorbents can be used in many industrial areas as catalysts, pigments, fillers, adsorbents, and ion exchangers (Gun’ko et al. 2004a).

Important processes related to the colloidal systems are stabilization or destabilization of the metal oxide aqueous suspensions in the presence of the polymer substances. Depending on conditions, the addition of the polymer can promote stable suspensions formation due to the steric or electrosteric stabilization of solid particles. On the contrary, the mineral oxide suspension undergoes destabilization as a result of the bridge flocculation or surface charge neutralization (Semenov 2008; Wisniewska 2011a). The mechanism of the process is strictly related to the polymer conformation at the solid–liquid interface and depends on the solution pH.

Because of significant importance of the aqueous suspensions stabilization/destabilization process and numerous potential applications of studied materials, it is important to determine the mechanism of the polymer adsorption on the mixed oxide surface as a function of the solution pH. Different structures as well as surface properties of the nanooxides in comparison to the adsorbents including of single oxides can contribute to the changes in nature of interactions between the macromolecules and the solid surface resulting in destabilization or an increase of the studied system stability. Changing conditions of the adsorption process have an essential impact on the macromolecular compound binding mechanism. Depending on the solution pH, the system is followed by a series of changes in the structure of both the mixed oxide surface groups and the polymer chain. Additionally, the stability mechanism was precisely determined due to application of the turbidimetric method. This allows to calculate the hydrodynamic parameters (the Turbiscan Stability Index value, the average aggregates diameter, and their sedimentation velocity), which is a high novelty element.

Assuming great demand for new materials with specific adsorption properties, the goal of this paper was

- Determination of the adsorbed amount of the nonionic polymer—polyvinyl alcohol (PVA) on the mixed oxide Al_2_O_3_–SiO_2_–TiO_2_ surface (AST 50) in the presence of the background electrolyte as a function of the solution pH,
- Investigation of the electrokinetic properties of AST 50 nanooxide and examination of the polymer molecular weight and hydrolysis degree influence on the adsorbent surface charge density as well as the zeta potential value,
- Study of the AST 50 suspension stability changes in the absence and presence of the nonionic PVA as a function of the solution pH,
- Propose of the stabilization/destabilization mechanism of the AST 50 mixed oxide aqueous suspension with the nonionic polymer.

## Materials and methods

In the paper, the ternary mixed oxide denoted as AST 50 was investigated. This material consisting of alumina (22 wt%), silica (28 wt%), and titania (50 wt%) was prepared by the chemical vapor deposition (CDV) method at the Institute of Surface Chemistry, National Academy of Sciences, Ukraine. The specific surface area of the adsorbent (*S*_BET_) was estimated using the nitrogen adsorption–desorption isotherms at 77.4 K (ASAP 2405 N, Micromeritics). The average diameter of primary particles was 51 nm (Zetasizer 3000, Malvern Instruments) (Wiśniewska et al. 2012b).

**Fig. 1:**
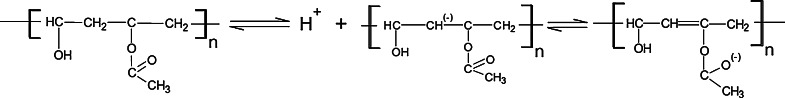
Formation of charge in the PVA chains [11]

As a macromolecular compound polyvinyl alcohol was used (Fluka). The polymer average molecular weights were 72,000 (PVA 72) and 100,000 Da (PVA 100). The hydrolysis degree for both substances was 97.5–99.5 % for PVA 72 and 86–89 % for PVA 100, respectively. These data were supplied by the producer. Polyvinyl alcohol belongs to the nonionic group of polymers. However, as a result of incomplete hydrolysis of the starting material, the polymer macromolecules contain a certain number of acetic groups, which are capable of ionization with the solution pH increase; Fig. 1 (Wiśniewska 2012b).

Adsorption measurements were conducted by the static method at the three pH values 3, 6, and 9 (±0.1). The polymer concentration after the adsorption process was estimated by using the calibration curve. To seven Erlenmeyer flasks, a suitable amount of the PVA stock solution (at a concentration of 500 ppm), the background electrolyte (0.01 mol/dm^3^ NaCl), and the doubly distilled water were added in order to obtain the polymer concentration range from 10 to 300 ppm. The solutions absorbance was determined on the basis of color reaction between the analyzed macromolecular compound, iodine, and boric acid. The absorbance was measured after 15 min using the UV–Vis Spectrometer (Cary 100, Varian) at a wavelength of 682 nm using the quartz cuvette. From the obtained results, the curves showing the dependence of absorbance on the PVA concentration were drawn up.

In order to investigate the adsorption of PVA on the AST 50 surface, 0.025 g of fumed oxide was added into the Erlenmeyer flasks containing 10 cm^3^ of polymer solution with fixed concentrations. After adjusting the appropriate pH values (by using 0.1 M NaOH or 0.1 M HCl), the samples were shaken in water bath (OLS 200, Grant) for 24 h. Then the sediments were centrifuged twice (MPW-223e, Centrifuge) and 5 cm^3^ of the polymer solution was taken for the analysis. All measurements were conducted at room temperature (≈25 °C) and in the presence of 0.01 mol/dm^3^ NaCl as a supporting electrolyte.

The potentiometric titrations were performed by using the following set of apparatus: Teflon vessel, thermostat RE 204 (Lauda), laboratory stirrer, glass and calomel electrodes (Beckman Instruments), pH meter (Radiometer), and automatic microburette Dosimat 765 (Metrohm) connected to the computer and printer. In order to obtain the mixed oxide potentiometric curves in the absence of the polymer, 50 cm^3^ of the supported electrolyte and 0.2 cm^3^ of 0.1 mol/dm^3^ HCl were placed into the thermostated Teflon vessel. The addition of acid provides the initial pH value in the range of 3–3.5. After reaching the equilibrium, 0.29 g of AST 50 was added. The obtained suspension was titrated by NaOH at a concentration of 0.1 mol/dm^3^. The potentiometric titrations in the presence of different molecular weights of PVA were conducted in the same way. The experiments were made in the polymer concentration range of 10–300 ppm. The surface charge density of the analyzed adsorbent in the absence and presence of PVA was calculated using the “Titr_v3” program written by W. Janusz.

The zeta potential measurements were made using Zetasizer Nano ZS (Malvern) coupled with the computer and automatic titrator. To obtain a solid suspension in the background electrolyte solution, 0.003 g of AST 50 was added to a 50 cm^3^ beaker containing a suitable amount of NaCl. The suspension was further sonicated for 3 min and the initial pH equal to 3 was fixed. The zeta potential measurement was carried out up to pH 10 and it was controlled by the computer software. During the experiment the AST 50 suspension was stirred by using a magnetic stirrer incorporated into a device. The samples with PVA were prepared in the same way, the polymer concentration range was 10–300 ppm.

Stability measurements were conducted using a turbidimeter Turbiscan Lab^Expert^ connected to the cooling module TLab Cooler and specialized computer software. This device possesses an electroluminescence diode which emits a collimated light beam (*λ* = 880 nm), passing through the studied suspension and two synchronized detectors. The transmission detector records the light passing through the sample at the angle of 0° in relation to the incident light direction. The second one is the backscattering detector, which registers the light scattered at the angle of 135°. The computer stores and processes the data.

The obtained results are presented in the form of transmissions and backscattering curves as a function of time. Low transmission level and high backscattering are characteristic of samples with considerable stability. The analysis of the turbidimetric data allows for the assessment of processes dynamics occurring in the sample during the measurement. The significant distances between both the transmissions and backscatter lines denote that in the system fast particles sedimentation takes place and the investigated suspension is unstable. Overlapping of the lines indicates an increased durability of the system. Moreover, due to the specialized computer software connected with Turbiscan, it was possible to calculate the TSI parameter (Turbiscan Stability Index) that is very useful in the evaluation of colloidal system stability. The TSI coefficient is calculated from the following formula:

1 $${\text{TSI}} = \sqrt {\frac{{\sum\nolimits_{i = 1}^{n} {(x_{i} - x_{\text{BS}} )^{2} } }}{n - 1}},$$

where *x*_*i*_ denotes the average backscattering for each minute of measurement, *x*_BS_ is the average value of *x*_*i*_, and *n* is the number of scans.

The coefficient is in the range from 0 (for highly stable systems) to 100 (in the case of very unstable suspensions). Based on the transmission and backscattering data, it was possible to determine the stability parameters such as the diameters of formed aggregates (particles, flocks) [μm] and the rate of particles (aggregates, flocks) migration [μm/min]. These data were calculated using the programs TLab EXPERT 1.13 and Turbiscan Easy Soft. The measurement lasted 15 h, during which data were collected every 15 min.

The studies involve determination of changes of aqueous suspensions stability in the absence and presence of the polyvinyl alcohol with the molecular weight of 100,000 Da. The samples without the polymer were prepared by adding 0.002 g of AST 50–20 cm^3^ of the supporting electrolyte at a concentration of 0.01 mol/dm^3^, then the suspensions were sonicated for 3 min. The next step was adjusting the required pH value of the samples. The suspensions containing PVA 100 were prepared in an analogs way. The polymer at a concentration of 100 ppm was added to the solid suspension after the sonication process. In order to investigate the effects of solution pH on the mixed ternary oxide suspension stability, the measurements were performed at pH equal to 3, 6, and 9.

## Results and discussion

**Fig. 2:**
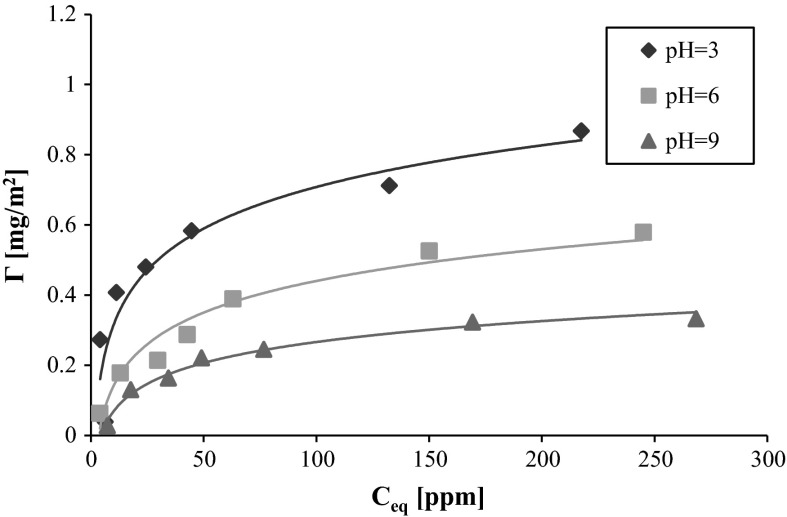
Adsorption isotherms of PVA 72 on the AST 50 surface at various solution pH values

**Fig. 3:**
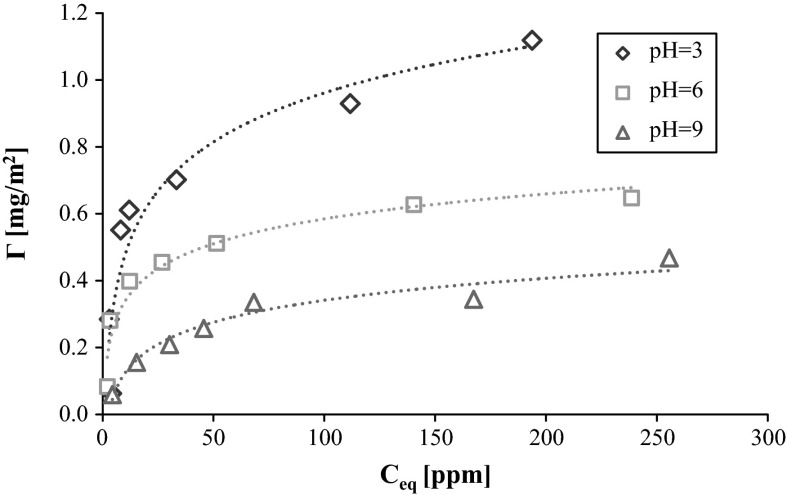
Adsorption isotherms of PVA 100 on the AST 50 surface at various solution pH values

The adsorption isotherms of different weights of polyvinyl alcohol on the AST 50 surface as a function of the three investigated solution pH values are shown in Figs. 2 and 3. The course of these dependencies indicates that the adsorption amount decrease is observed when the solution pH grows. This is related to the mixed oxide surface charge changes. The analysis of AST 50 composition leads to the conclusion that at pH 3 the majority of the nanooxide surface groups are positively charged. The hydroxyl groups occurring on the oxide materials surface exhibit the amphoteric nature. For the aqueous solutions, the most important in the charge formation process are hydrogen and hydroxyl ions which can interact with the surface active groups in the following ways (Janusz et al. 1997):

2 $$\equiv {\rm MOH}_{2}^{ + } \leftrightarrow \equiv {\rm MOH} + {\rm H}^{ + }$$

3 $$\equiv MOH + OH^{ - } \leftrightarrow \equiv MO^{ - } + H_{2} O$$

4 $$\equiv {\rm M}_{1} - {\rm O}({\rm H}) - {\rm M}_{2} \equiv \leftrightarrow \equiv {\rm M}_{1} \left( {\rm O}^{ - } \right) - {\rm M}_{2} \equiv + \,{\rm H}^{ + },$$

where M denotes the metal cation (Ti, Si, Al) and M_1_ ≠ M_2_. Equations (2) and (3) refer to the equilibrium state achieved under the acidic and alkaline conditions, respectively. The last one concerns the bridging hydroxyl groups (Brönsted acids). They can be easily deprotonated and are capable of electrostatic or hydrogen bond formation (Semenov 2008). Furthermore, on the adsorbent surface can occur incompletely O-coordinated metal atoms such as Al, Ti, or Si (Lewis acids) and other active centers e.g., terminal hydroxyls (Gun’ko et al. 2010b). The above-mentioned surface groups are responsible for the formation of strong adsorption complexes with polymer macromolecules. It is clearly visible from the presented equations that the hydroxyl ions present in the solution have a distinct impact on the nature of the metal oxide particles –negative charges appear gradually on their surface with pH rise. The solution pH value, when the number of positively and negatively charged active surface groups is equal, is named pH_pzc_ (pzc—point of zero charge). This value is specific for a given material and depends on the mineral oxide structure (Kosmulski 2009).

**Fig. 4:**
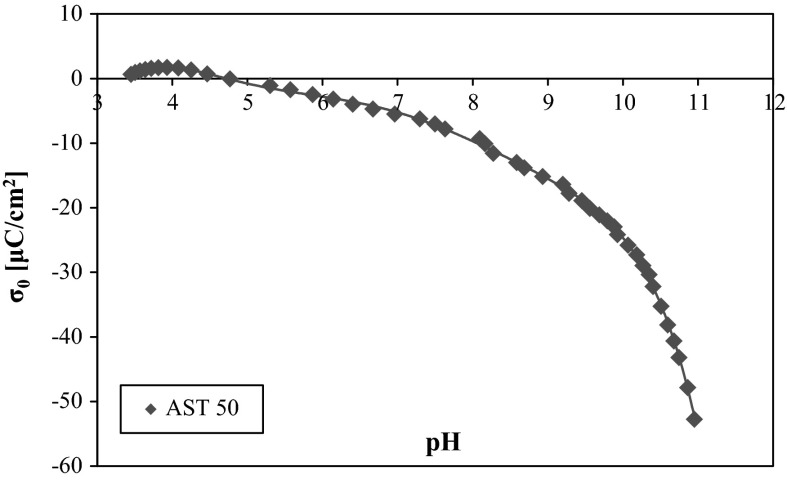
Surface charge density of AST 50 in the presence of background electrolyte

The analysis of the AST 50 surface charge density (in the presence of NaCl as a supporting electrolyte, Fig. 4) indicates that at pH 3 most of the nanooxide surface active groups are positively charged. The point of zero charge is equal to about 4.7. Above this value, the mixed oxide surface carries a negative charge. It should be mentioned that in the whole solution pH range of the solution, certain number of the neutral surface groups (such as Al–OH, Si–OH, and Ti–OH) is present on the AST 50 surface. They are mainly responsible for the hydrogen bond formation.

Due to the fact that the studied polymer has the functional groups capable of negative charges formation in its structure, the PVA macromolecules can interact electrostatically with the oppositely charged adsorbent surface centers as it takes place at pH 3. Under these conditions, the majority of the solid surface groups are positively charged, especially those originating from alumina and titania. However, because of a small number of the ionized acetic groups, the hydrogen bridges formed between the PVA and the adsorbent hydroxyl groups play a dominant role in the polymer adsorption process. Additionally, the hydrophobic interactions between the adsorbed polymer macromolecules affect the process. Moreover, a low ionization degree of the PVA chains promotes the formation of more compact polymer coil conformation on the solid surface. As a result, the PVA macromolecules occupy less space per unit area of the mixed oxide and the increase in adsorption is observed.

At pH 6, despite the increase in the PVA chains ionization degree, the polymer adsorption is considerably reduced compared to the results achieved at pH 3. It is related to a decrease in the number of the positively charged surface groups capable of the electrostatic interactions with the acetic groups belonging to the polymer chains. Under these pH conditions, the PVA adsorption is most likely driven by the hydrogen bond formation and the dispersive forces.

Further pH growth causes a significant increase in the number of negative charges on the mixed oxide surface and as a consequence, rise of repulsion forces between the solid particles and the polymer macromolecules. Therefore, PVA can undergo adsorption only through formation of the hydrogen bonds by the hydroxyl groups occurring in the macromolecules. Moreover, the higher ionization degree is responsible for the PVA chains development (as a result of the mutual repulsion of the same charges). In this situation, the polymer coils need more space on the solid surface blocking the access to the other adsorbent active centers available for the PVA chains present in the solution. This is a main reason for the polyvinyl alcohol adsorption amount reduction at pH 9.

From the comparison of Figs. 2 and 3, one can notice that the PVA adsorption increases with the polymer molecular weight growth in the whole solution pH range. It follows from a greater affinity of the higher molecular weight macromolecular compound for the AST 50 surface. What is more, the hydrolysis degree strongly influences the analyzed polymer adsorption properties. PVA 72 contains on the average about 1.5 % of the acetic groups in the chains, whereas in the PVA 100 macromolecules the number of functional groups capable of negative charge formation is about 10 times larger. The studies carried out earlier revealed that the negatively charged acetic groups undergo a preferential adsorption on the mineral oxide surface compared to the neutral hydroxyl groups which are capable of only hydrogen bridges formation (Chibowski et al. 2000).

**Fig. 5:**
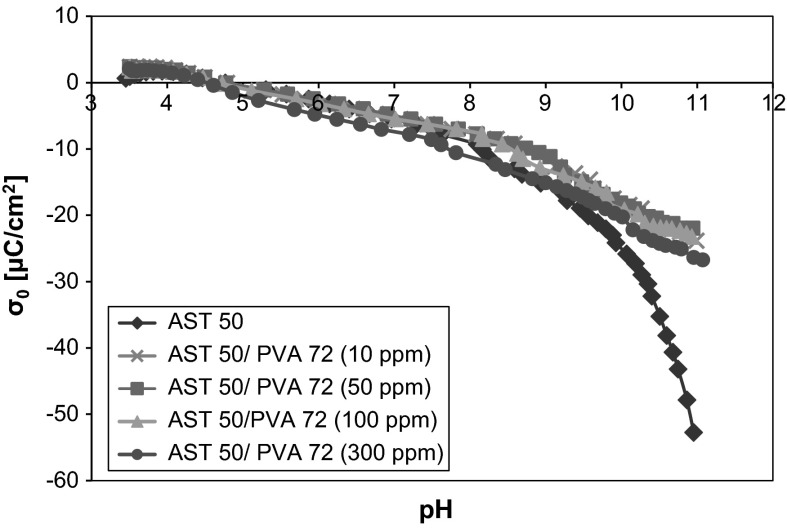
Density of the AST 50 surface charge with and without PVA 72 of different concentrations

**Fig. 6:**
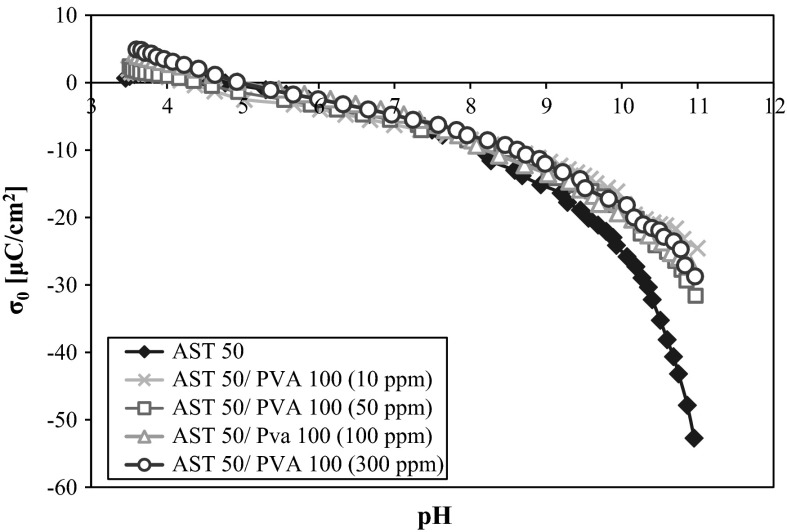
Density of the AST 50 surface charge with and without PVA 100 of different concentrations

Analyzing the influence of the nonionic PVA on the potentiometric titration curves (Figs. 5 and 6), it should be noticed that the polymer adsorption hardly changes the AST 50 surface charge density in the pH range from 3 to 8. Above that a slight increase in the mixed oxide *σ*_0_ values (compared to the surface charge density without the macromolecular compound) is observed. It leads to the conclusion that the reason for such a behavior is the positive charge induction on the AST 50 particles on account of the interactions between the PVA macromolecules and the solid surface active groups. This effect does not occur distinctly at the lower pH values due to a small number of the ionized acetic groups located near the solid surface. As it was mentioned before, under these conditions the hydrogen bonds are responsible for the PVA macromolecules binding, but this type of interactions does not influence the surface charge density values changes. The increase in the polymer ionization degree (the resulting expanded conformation of the chains) contributes to a larger number of the electrostatic interactions between the negatively charged functional groups and the adsorbent surface.

**Fig. 7:**
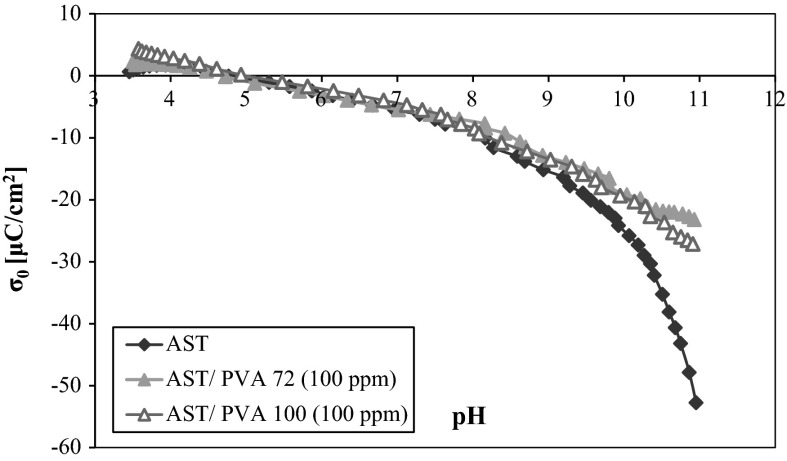
Comparison of the potentiometric curves for different weights of PVA at a concentration of 100 ppm

The potentiometric data obtained for the systems containing PVA at different concentrations (but the same molecular weight) point out that the polymer concentration has no essential impact on the AST 50 surface charge density. The curves are almost overlapping for both PVA 72 and PVA 100. This comes from the presence of an insignificantly larger number of the polymer functional groups with the concentration growth. Some conclusions on the influence of the PVA molecular weight on the AST 50 *σ*_0_ values can be drawn from the dependencies present in Fig. 7. The effect associated with the increase in the adsorbent surface charge density observed above pH 8 is considerably smaller for the higher molecular weight polymer. Such a behavior can be explained by the presence of a larger number of the ionized acetic groups belonging to the PVA 100 chains (with regard to PVA 72). The increase in the polymer charged functional groups contributes to a higher number of the negative charges located near the solid surface. Hence, the influence of the positive surface charge induction is smaller on account of the negatively charged acetic groups presence in the AST 50 by-surface layer.

**Fig. 8:**
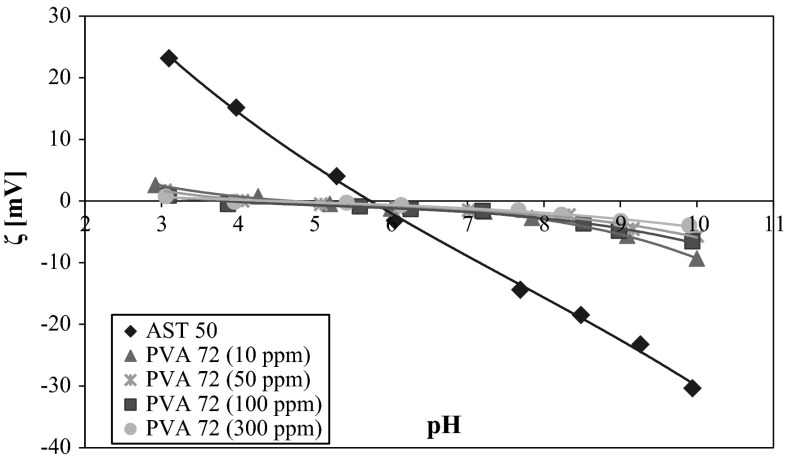
AST 50 zeta potential in the absence and presence of PVA 72 at different concentrations

**Fig. 9:**
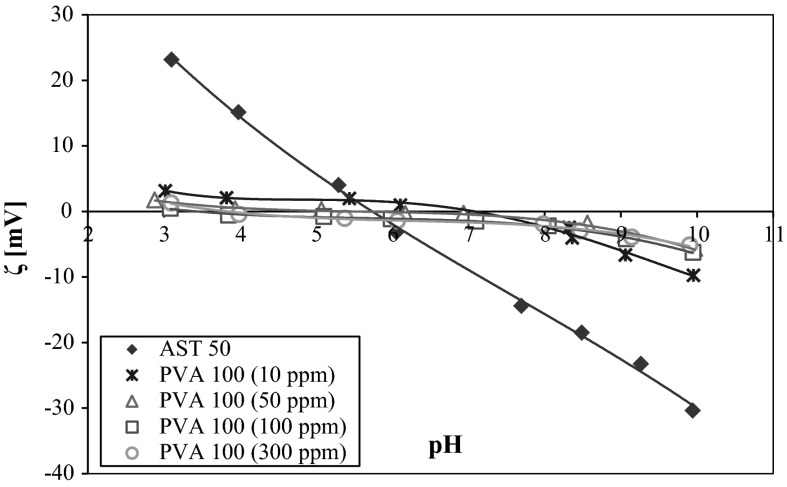
AST 50 zeta potential in the absence and presence of PVA 100 at different concentrations

In order to make a comprehensive electrokinetic analysis of the studied system, the AST 50 zeta potential measurements were carried out in the absence and presence of the nonionic polymer (Figs. 8 and 9). It can be clearly seen from the data presented in Fig. 8 that the values of the mixed oxide electrokinetic potential without the nonionic polymer are positive in the pH range from 3 to 5.8 and negative under the other pH conditions. At pH 5.8, the isoelectric point (pH_iep_) is reached where the total charge of the diffusion layer is equal to zero. Changes in the zeta potential in the presence of polymer may be caused by three different effects:

- the presence of the charges coming from the polymer-dissociated functional groups in the by-surface layer of the solid,
- the shift of the slipping plane by the macromolecules adsorbed on the metal oxide surface,
- the displacement of the counter-ions in the Stern layer as a result of the polymer adsorption (Vincent 1974).

From the course of the AST 50 zeta dependencies obtained in the presence of both lower and higher molecular weight polymer, it was found that PVA attendance considerably influences on the mixed oxide electrokinetic properties changes. In the pH range 3–5.8, the AST 50 zeta potential is reduced, whereas above the pHiep point the PVA macromolecules adsorption leads to the marked zeta growth compared to the values achieved without the polymer. The most important reason for this phenomenon are changes in the polymer binding mechanism on the colloidal particles surface. In the pH range from 3 to 6, the number of negatively charged acetic groups present in the chains is small. As a result, under these conditions the polymer adsorption is driven by the hydrogen bond formation. In such a case on the mixed oxide surface, the compact adsorption layer of a small thickness is created (the polymer coils are closely packed). The adsorbed polymer chains shift the slipping plane and cause the zeta potential reduction. With the solution pH rise, the macromolecular compound adsorption decreases because of the repulsion forces occurring between the PVA macromolecules and the adsorbent surface. The polymeric chains extension on the solid surfaces promotes the formation of more stretched polymer coil structure rich in numerous loops and tails. This is related to the larger slipping plane shift by the adsorbed polymer macromolecules. Moreover, at pH above 6 on the AST 50 surface there are mainly present negatively charged groups which are associated with the supporting electrolyte cations. But the PVA chains undergoing the adsorption process interact with the positively charged adsorbent groups inducing the opposite counter-ions displacement toward the diffusion part of electrical double layer. As a consequence, the AST 50 particles zeta potential values growth is observed compared to the results obtained without the nonionic polymer. As in the case of the potentiometric data, there are no significant differences in the ζ potential values with the PVA concentration growth. The most important reason for this phenomenon is a very small increase in the number of functional groups in the PVA chains with the concentration increase.

A lack of visible influence of the analyzed polymer molecular weight on the zeta potential can be explained by the mutual removal of the two effects: the slipping plane shifting and the counter-ions displacement from the Stern plane toward the diffusion layer. The lower molecular weight polymer on the AST 50 surface adopts a flat conformation, so the PVA 72 macromolecules can interact with numerous adsorbent surface active groups. This leads to the situation in which the number of counter-ions removed from the Stern plane increases. Simultaneously, the contribution of the slipping plane shifting by a less densely packed polymer adsorption layer is smaller. Due to the existence of a larger number of the acetic groups in the adsorbed PVA 100 chains, on the AST 50 particles surface the adsorption layer rich in loops and tails is formed. Under these conditions the polyvinyl alcohol segments interact with a smaller number of the adsorbent surface groups leading to the situation in which a number of the counter-ions displaced from the by-surface layer decreases. In addition, the formation of a thicker adsorption layer promotes greater slipping plane shifting. The total contribution of these two mentioned effects to the zeta potential value is approximately the same for both polymers. For this reason, there is no observed substantial influence of the studied polymer molecular weight on the electrokinetic potential changes.

**Fig. 10:**
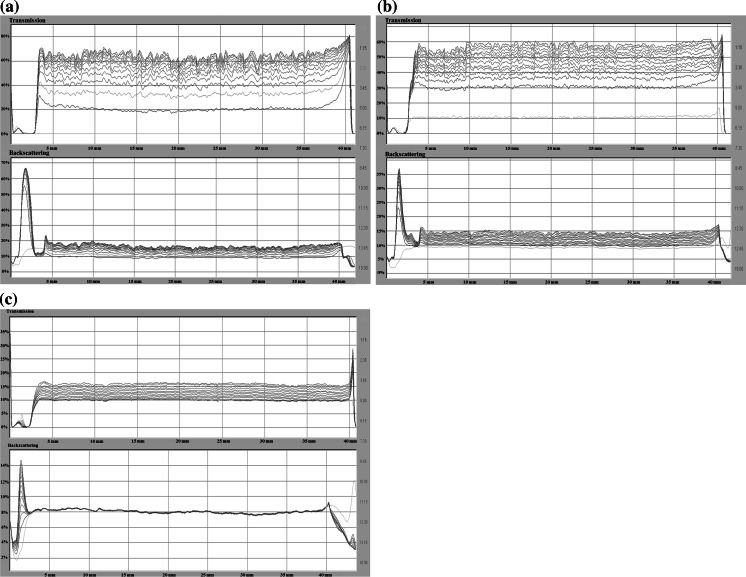
Transmission and backscatter curves for AST 50 suspensions: **a** pH 3, **b** pH 6, **c** pH 9

The transmission and backscatter curves obtained for the AST 50 suspension without the polymer recorded at three studied pH values (3, 6, and 9) are shown in Figs. 10 (a–c). As one can notice from the presented data, at pH 3 the AST 50 suspension exhibits considerably lower stability in relation to the other investigated pH values in the two systems. This is confirmed by the highest transmission level as well as the relatively high TSI parameter value (44.58; Table 1). Furthermore, the distance between the successive scans increases (as a function of time) which is associated with the suspended particles sedimentation. The lack of the system durability can be explained by the electrostatic attraction forces between the positively and negatively charged surface groups belonging to the different AST 50 particles.

**Table 1:** TSI values for the AST 50 mixed oxide suspensions in the absence and presence of polyvinyl alcohol (*c* = 100 ppm) at three pH values

System	TSI		
	pH = 3	pH = 6	pH = 9
AST 50	44.58	32.38	2.59
AST 50/PVA 100 (100 ppm)	7.47	17.78	3.55

At pH 6, a slight decrease in the transmission compared to pH 3 is observed. However, the lines corresponding to each scan are still at a certain distance from each other. Nevertheless, the system becomes still relatively unstable. The main reason for such a behavior is the fact that under these conditions the solid isoelectric point is reached. This indicates that the mixed oxide particles diffusion layers are practically free from the electric charges, which facilitates their aggregation.

A sharp decrease in the transmission level in the case of the mixed oxide suspension at pH 9 is related to significant durability of the studied system. The backscattering overlapping lines indicate the formation of a suspension characterized by high stability, which remains practically constant for the whole measurement time (TSI = 2.59). Such a behavior can be explained by numerous negative charges on the solid surface leading to effective particles repulsion.

**Fig. 11:**
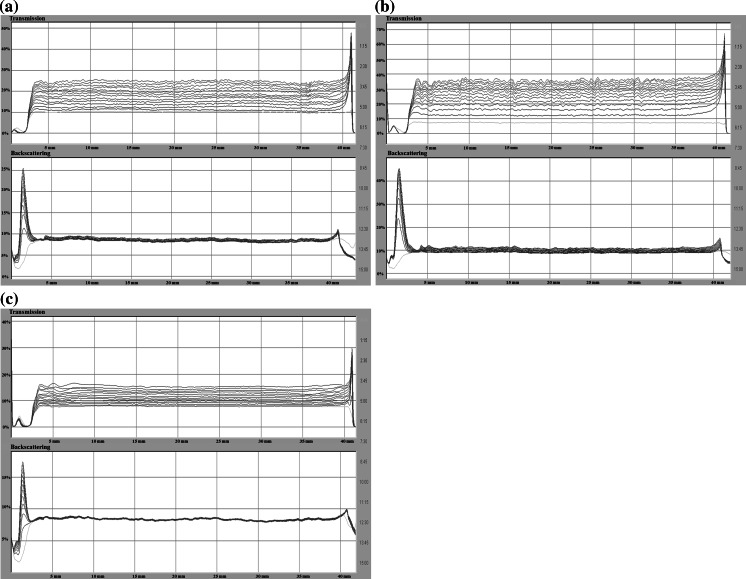
Transmission and backscatter curves for the AST 50 suspensions containing PVA 100 (at a concentration of 100 ppm): **a** pH 3, **b** pH 6, **c** pH 9

**Fig. 12:**
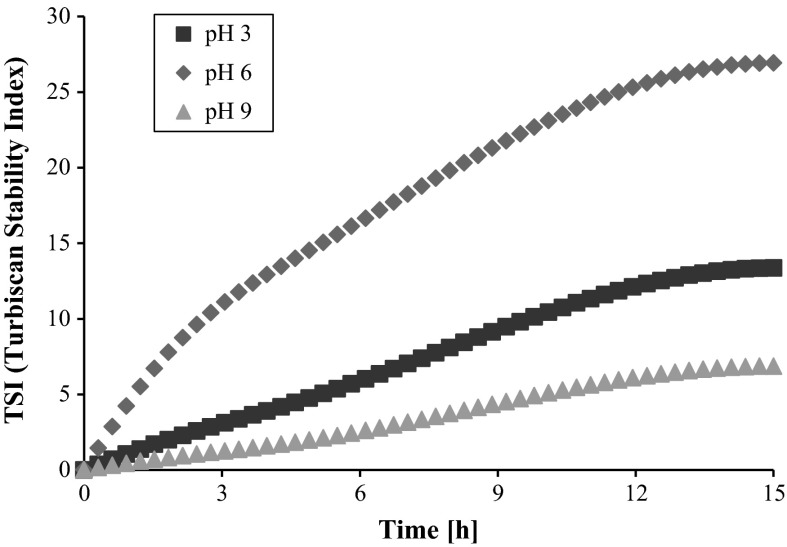
The TSI changes for AST 50 suspensions in the presence of PVA 100 at a concentration of 100 ppm as a function of time

**Fig. 13:**
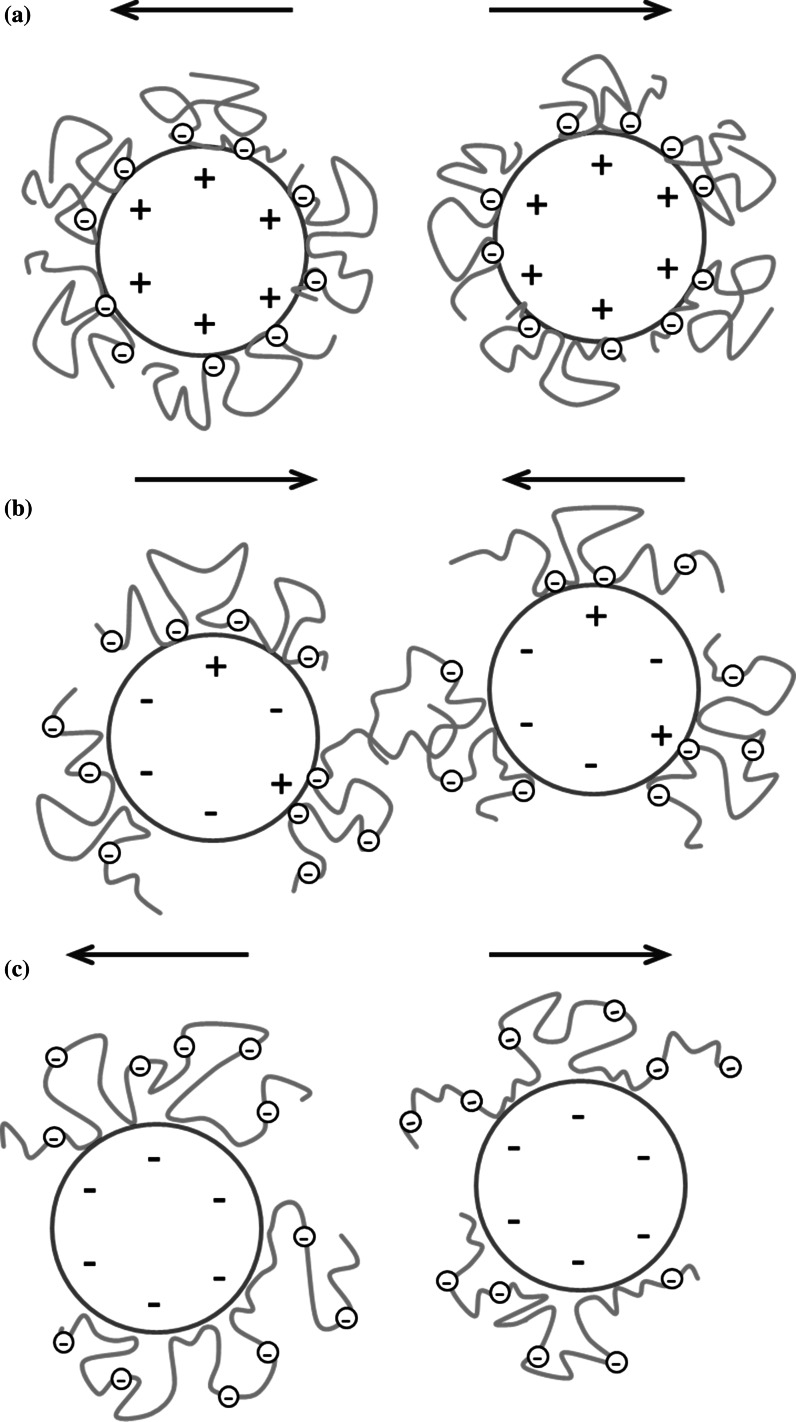
Schematic representation of stabilization (destabilization) mechanism of AST 50 suspension in the presence of PVA 100 at: **a** pH 3, **b** pH 6, **c** pH 9

The addition of the nonionic polymer (PVA 100) has an essential influence on the AST 50 suspension stability (Figs. 11 (a–c) and 12). As follows from the turbidimetric data analysis, the system stability growth is observed for the samples at both pH 3 and 6—the transmission intensity was reduced by half compared to the values obtained without PVA under the same conditions. The TSI coefficient value is also lower. In turn, at pH 9 there are no significant stability changes associated with the PVA 100 presence (TSI = 3.55). The proposed stabilization (destabilization) mechanism for the AST 50 suspensions in the presence of PVA 100 is shown in Fig. 13.

A significant increase in the system stability at pH 3 follows from the strong polymer adsorption at the solid–liquid interface. On account of a low PVA ionization degree, the closely packed adsorption layer containing strongly coiled PVA macromolecules is formed on the AST 50 surface. This polymer layer structure ensures the mixed oxide suspension steric stability (TSI = 7.47).

The durability growth of the system containing PVA 100 at pH 6 (in relation to the suspension in the polymer absence) is a result of the acetic groups ionization and development of the chains adsorbed on the solid surface. Under these conditions the solid particles are also stabilized electrostatically due to the presence of numerous negatively charged functional groups in the PVA 100 macromolecules. This mechanism is described as electrosteric stabilization. The higher TSI value (rise by 10 units) as well as the larger size of the formed aggregates (Table 3) indicates that the sample containing nonionic polymer exhibits lower stability with regard to the suspension at pH 3. The reason for this situation can be appearance of single polymer bridges between the PVA chains adsorbed on two different oxide particles.

At pH 9, PVA 100 does not influence on the mixed oxide suspension stability. Both the position of the transmission and backscatter curves as well as the TSI coefficient value obtained for the sample with the polymer are practically unchanged in comparison to the data in the PVA absence. Under these conditions the PVA adsorption is the lowest in comparison to strong polymer macromolecules ionization. The polymer chains structure development toward the bulk solution leads to appearance of the forces related to the steric effects, while the increase in the number of negative charges is connected with the presence of the electrostatic repulsion forces between the particles covered with PVA (electrosteric stabilization).

Based on the transmission and backscattering data, it was possible to determine the stability parameters such as the diameters of formed aggregates (particles, flocks) [μm] and the rate of particles (aggregates, flocks) migration [μm/min]. These data were calculated using the programs TLab EXPERT 1.13 and Turbiscan Easy Soft. The particle velocity rate was calculated on the basis of the multiple light scattering theory. The particles diameter were determined using the general law of sedimentation, which is Stokes’ law extended to the concentrated dispersions (Snabre and Mills 1994):

5 $$V(\phi ,d) = \frac{{\left| {\rho_{\text{p}} - } \right.\left. {\rho_{\text{c}} } \right| \cdot g \cdot d^{2} }}{{18 \cdot v \cdot \rho_{\text{c}} }} \cdot \frac{[1 - \varphi ]}{{\left[ {1 + \frac{4.6\varphi }{{(1 - \varphi )^{3} }}} \right]}},$$

where *V* is the particles migration velocity [µm/min], *ρ*_c_ is the continuous phase density [kg/m^3^], *ρ*_p_ is the particle density [kg/m^3^], *d* is the particle mean diameter [µm], *v* is the continuous phase viscosity [cP], and *φ* is the volume of the dispersed solid fraction [%].

The data calculated from the turbidimetric measurements (as a function of the studied solution pH) are presented in Tables 2 and 3. The thickness of the formed sediment in both the absence and presence of the macromolecular compound reduces as the solution pH rises. It originates from the studied suspension stability growth. Another conclusion that can be drawn during analyzing these results is that the adsorption of PVA 100 is reflected by a decrease in the sediment layer thickness created on the measuring vial bottom.

**Table 2:** Thickness of sediment and the clear layer formed in the studied systems depending on the solution pH

System	Thickness of sediment [mm]			Thickness of the clear layer [mm]		
	pH = 3	pH = 6	pH = 9	pH = 3	pH = 6	pH = 9
AST 50	1.11	0.75	0.66	Lack	Lack	0.80
AST 50/PVA 100 (100 ppm)	0.81	0.58	0.46	0.8	1.0	1.0

**Table 3:** Average velocity of aggregates sedimentation (V) and average thickness of sediment in the AST 50 suspension

System	*d* [μm]			*V* [μm/min]		
	pH = 3	pH = 6	pH = 9	pH = 3	pH = 6	pH = 9
AST 50	0.65	0.71	0.22	41.49	48.12	12.22
AST 50/PVA 100 (100 ppm)	0.67	0.74	0.46	44.07	53.27	20.77

As one can notice, in the AST 50 suspensions without the polymer the largest aggregates are formed at pH 6, while the smallest size is achieved in the alkaline solution pH. The reason for this situation is the forces responsible for the mutual attraction (pH 6) or repulsion (pH 3 and 9) of the suspended particles. The addition of PVA 100 causes the average aggregates size increase on account of the polymer macromolecules adsorption. In relation to the solution pH value, the interactions between the adsorption layers belonging to the different AST 50 particles change leading to the mixed oxide aggregates diameter changes. As in the case of the samples without the polymer addition, the largest flocs in the presence of PVA are formed at pH 6. This is most likely driven by the appearance of single polymer bridges formed due to the adsorption of one PVA macromolecule on two (or more) solid particles (as a result of the electrostatic interactions and the hydrogen bond formation). Smaller aggregates sizes were obtained for the colloidal suspensions containing polyvinyl alcohol at both pH 3 and 9. At the acidic solution pH, the suspensions exhibit steric stabilization, whereas under the alkaline conditions stabilization follows from the electrostatic effects. The average sedimentation velocities calculated for all studied systems depend on the aggregate size—the larger their diameter, the greater the rate of sedimentation.

## Conclusions

The nonionic polyvinyl alcohol adsorption on the mixed oxide Al_2_O_3_–SiO_2_–TiO_2_ (AST 50) surface strictly depends on the solution pH values. The maximum of the PVA adsorption amount is reached at pH 3, whereas the fewest polymer macromolecules were bound to the adsorbent surface in the alkaline environment. The observed decrease in the amount of adsorbed PVA macromolecules with the solution pH growth is associated with the electrostatic repulsion forces present in the studied system. The more basic solutions, the more negatively charged AST 50 surface is. The increase in the ionization degree of the acetic groups belonging to the polymer chains leads to acquisition of more developed structure of the PVA macromolecules. The electrostatic repulsion forces occurring between the identically charged mixed oxide particles and the polymer chains are responsible for the reduction of the polyvinyl alcohol adsorption size.

The potentiometric data show that the pH_pzc_ value of the mixed ternary oxide is equal to 4.7. Due to the nonionic character of the analyzed macromolecular compound, the polymer attendance has an insignificant effect on the AST 50 surface charge density. A slight growth of the adsorbent surface charge above pH 8 is related to the induction of positively charged surface groups. This process follows from the adsorption of the PVA macromolecules containing a small number of the ionized acetic groups (which are the source of negative charge). The polymer concentration increase does not cause considerable solid surface charge density changes. There are also no meaningful changes in the *σ*_0_ values as a function of PVA molecular weight—in the presence of PVA 100 the surface charge density of AST 50 assumes a smaller value compared to PVA 72. This is a consequence of a larger content of the acetic groups in the higher molecular weight polymer chains.

Adsorption of PVA strongly affects the AST 50 particles zeta potential value. In the presence of the nonionic polymer, reduction of the electrokinetic potential values (in the range of solution pH from 3 to 6) is observed. The opposite situation takes place from pH 6–10 where the *ζ* potential increases. A decrease in the zeta potential is related to the slipping plane shifting by the adsorbed polymer chains. In turn, the zeta growth in the PVA presence results from displacement of the counter-ions from the Stern plane toward the diffusion layer. The total contribution of these two effects is responsible for the experimentally observed electrokinetic potential values of the solid particles covered with the polyvinyl alcohol macromolecules.

The stability measurements have shown that the AST 50 suspensions in the presence of the background electrolyte are the least stable at pH 3 and 6. In turn, in an alkaline medium the mixed oxide suspensions exhibit the highest durability. Low stability at pH 3 follows from the fact that on the AST 50 particles surface there is accumulated a small amount of positive charges. The electrostatic forces between the positively and negatively charged surface groups of different particles cause destabilization of the studied system. With the solution pH increase, the solid surface becomes negative. At pH 9, the number of the negatively charged groups present on the adsorbent particles surface is sufficient to provide the long-term stability of AST 50 suspension.

The addition of PVA 100 significantly improves the suspension stability at both pH 3 and 6, at the higher pH value the polymer presence does not influence the system durability. In the acidic solution, colloidal particles are sterically stabilized due to the high adsorption of the strongly coiled polymer macromolecules. With the pH growth, the macromolecular compound chains adopt more developed conformation (due to the increase in the acetic groups ionization). This leads to the formation of the polymer adsorption layer capable of improving the electrosteric stabilization.

## References

[CR1] Amjad Z (2002). Water soluble polymers solution properties and applications.

[CR2] Briscoe B, Luckham P, Zhu S (2000). The effects of hydrogen bonding upon the viscosity of aqueous poly(vinyl alcohol) solutions. Polymer.

[CR3] Cai W, Gupta RB (2002) Hydrogels. In: Kirk-Othmer (ed) Encyclopedia of chemical technology. Wiley, New York, pp 729–759

[CR4] Chibowski S, Paszkiewicz M, Krupa M (2000). Investigation of the influence of the polyvinyl alcohol adsorption on the electrical properties of Al_2_O_3_—solution interface, thickness of the adsorption layers of PVA. Powder Technol.

[CR5] Farrokhpay S (2009). A review of polymeric dispersant stabilisation of titania pigment. Adv Colloid Interface.

[CR6] Grządka E (2011). Influence of surfactants on the structure of the adsorption layer in the system: carboxymethylcellulose/alumina. Mater Chem Phys.

[CR7] Gun’ko VM, Zarko VI, Mironyuk IF (2004). Surface electric and titration behaviour of fumed oxides. Colloids Surf A.

[CR8] Gun’ko VM, Yurchenko GR, Turov VV (2010). Adsorption of polar and nonpolar compounds onto complex nanooxides with silica, alumina and titania. J Colloid Interface Sci.

[CR9] Janusz W, Kobal I, Sworska A, Szczypa J (1997). Investigation of the electrical double layer in a metal oxide/monovalent electrolyte solution system. J Colloid Interface Sci.

[CR10] Kosmulski M (2009). Surface charging and points of zero charge. Surfactant science series vol. 145.

[CR11] Liufu S, Xiao H, Li Y (2005). Adsorption of poly(acrylic acid) onto the surface of titanium dioxide and the colloidal stability of aqueous suspension. J Colloid Interface Sci.

[CR12] Mauer KH (2004). Detergent proteases. Curr Opin Biotech.

[CR13] Moody G (1992). The use of polyacrylamides in mineral processing. Min Eng.

[CR14] Semenov AN (2008). Theory of colloid stabilization in semidilute polymer solutions. Macromolecules.

[CR15] Snabre P, Mills P (1994). Settling of a suspension of hard spheres. Europhys Lett.

[CR16] Vincent B (1974). The effect of adsorbed polymers on dispersion stability. Adv Colloid Interface.

[CR17] Wiśniewska M (2011). A review of temperature influence on adsorption mechanism and conformation of water soluble polymers on the solid surface. J Dispers Sci Technol.

[CR18] Wiśniewska M, Chibowski S, Urban T (2012). Effect of the type of polymer functional groups on the structure of its film formed on the alumina surface—suspension stability. React Funct Polym.

